# Surface Irregularity Factor as a Parameter to Evaluate the Fatigue Damage State of CFRP

**DOI:** 10.3390/ma8115407

**Published:** 2015-11-11

**Authors:** Pablo Zuluaga-Ramírez, Malte Frövel, Tomás Belenguer, Félix Salazar

**Affiliations:** 1 Instituto Nacional de Técnica Aeroespacial (INTA), Carretera de Ajalvir Km 4, Torrejón de Ardoz 28850, Spain; frovelm@inta.es (M.F.); belenguer@inta.es (T.B.); 2 Department of Applied Physics, Escuela Técnica Superior de Ingenieros de Minas y Energía, Universidad Politécnica de Madrid, C/ Ríos Rosas 21, Madrid 28003, Spain; felixjose.salazar@upm.es

**Keywords:** fatigue damage, composites materials, CFRP, non-destructive test, non-contact inspection, optical inspection, spectrum fatigue loads, irregularity factor

## Abstract

This work presents an optical non-contact technique to evaluate the fatigue damage state of CFRP structures measuring the irregularity factor of the surface. This factor includes information about surface topology and can be measured easily on field, by techniques such as optical perfilometers. The surface irregularity factor has been correlated with stiffness degradation, which is a well-accepted parameter for the evaluation of the fatigue damage state of composite materials. Constant amplitude fatigue loads (CAL) and realistic variable amplitude loads (VAL), representative of real in- flight conditions, have been applied to “dog bone” shaped tensile specimens. It has been shown that the measurement of the surface irregularity parameters can be applied to evaluate the damage state of a structure, and that it is independent of the type of fatigue load that has caused the damage. As a result, this measurement technique is applicable for a wide range of inspections of composite material structures, from pressurized tanks with constant amplitude loads, to variable amplitude loaded aeronautical structures such as wings and empennages, up to automotive and other industrial applications.

## 1. Introduction

The knowledge of the fatigue damage state of structures made of composite material such as carbon fiber reinforced polymer (CFRP) is essential to make the next step in the optimization of composite structures in the aeronautic industry and in general industrial applications. Nowadays, fatigue damage is not an essential point in maintenance of aeronautic CFRP structures because these are normally loaded far below their fatigue resistance. However, but for future highly optimized structures, estimating the fatigue damage will become an important issue.

Conventional techniques to evaluate the fatigue state of an aircraft structure are based on measurements of structural loads throughout the service life by electric strain gauge sensors, which present some difficulties. One of them is that these sensors are affected by the extreme environmental flight conditions and by the fatigue loads, in such a way that they have an elevated probability to fail and require an exhaustive maintenance program. A second disadvantage is that the stiffness degradation of the composite materials due to the accumulated damage could lead to non-realistic stress-strain relation of the strain gauge sensors. A third disadvantage is that the accumulated fatigue damage determined by a load history is conventionally calculated by linear damage accumulation models (such as Palmgren-Miner) [1]. Experimental studies show that this rule leads to inaccurate and non-conservative predictions for composite materials under realistic variable amplitude loads [2,3,4].

There are models developed for fatigue damage accumulation of composite materials, including strength and stiffness degradation. These models are based on fitting experimental values [2], but, due to the complexity of the damage mechanisms, they are only applicable for explicit conditions of loads and materials.

The most classic fatigue damage metric is the strength degradation of the material, commonly known as residual strength [2]. When the residual strength becomes equal or lower than the maximum stress applied during cycling, the structure is prone to fail. Although the residual strength is the best metric to determine the damage state of a structure, in actual structures it cannot be measured by non-destructive evaluations.

Other classic fatigue damage metric of CFRP, and an alternative to the residual strength, is the stiffness degradation. Several authors have studied stiffness degradation and its relation with residual strength [5,6,7]. The advantage is that the stiffness can be measured with non-destructive methods, but in general it requires the application of a specific load to the structure with a dedicated test setup that is complicated to achieve in aircraft structures.

These difficulties have created the need to assess phenomenological methods [1] such as acoustic emissions [8], digital image correlation [9,10], thermography [10,11], X ray tomography [12], electrical resistance [13], and analysis of the surface [14,15,16], among others.

Previous studies by the authors have shown that changes in the surface topography of CFRP, quantified by the roughness magnitude, could be an indicator of the accumulated fatigue damage [14,15,16]. Other authors have studied techniques for evaluating the fatigue damage by means of the surface assessment in metal structures, which undergo a surface transition related to metallurgical effects of their crystal structure [17,18]. For CFRP, the change of the surface starts at the beginning of the life of the structure with matrix micro-cracks parallel to the reinforcing carbon fibers. With increasing fatigue cycles, the cracks become bigger and tend to produce local delaminations in sub-surface layers changing the surface topography significantly. Results of previous works by the authors with specimens cycled with constant amplitude loads (CAL) show that the changes of the surface roughness are correlated with the stiffness degradation [14,15], which is a classical metric of fatigue damage. A further study by the authors, which was focused on the evaluation of the methodology with realistic variable amplitude loads (VAL), shows that the agreement between surface roughness magnitude and stiffness degradation is independent of the type of load applied [16]. As mentioned by Sonsino [19], due to the absence of effective cumulative damage, models that predict the life of components under VAL using CAL test data or variable amplitude load tests (VAL) are useful and necessary to understand the behavior of the material under real in-service conditions.

The present work evaluates a different approach to assess the changes on the surface topography as a consequence of fatigue damage. The assessment is done in the frequency domain by determining the Power Spectral Density (PSD) of the topography profile, by evaluating the Irregularity Factor (*I*). This factor was first introduced by Rice [20,21] and is commonly employed to estimate the fatigue life of structures taking into account the PSD of the load history [19,22].

### Surface Irregularity Factor

**Figure 1:**
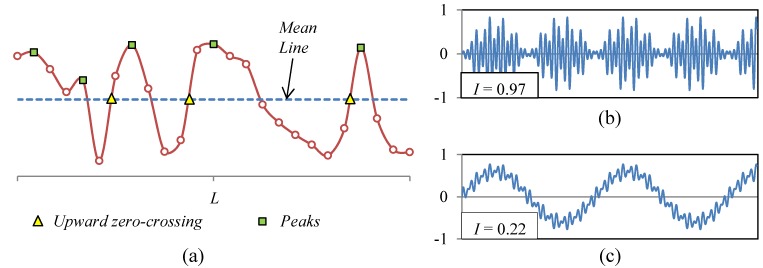
(**a**) Topographic profile with the theoretical points for calculating the irregularity factor; (**b**) Surface with a narrow band, *I* = 0.97; (**c**) Surface with a broad band, *I* = 0.22.

The irregularity factor estimates statistically the relation between the numbers of peaks in the profile and the number of upward zero-crossings (see Figure 1a). The *I*-Factor provides information of the shape of the surface. The mathematical procedure to determine the *I*-Factor starts with the fast Fourier transform algorithm (FFT). The topographic profile is defined by the discrete function *Z*(*x_j_*) that defines the height of the point *x_j_* respect to the mean line. The discrete function of the profile into the frequency domain is *Z*(ξ*_k_*), which is given by the Equation (1):

(1) $$Z({\text{ξ}}_{k})=\Delta x\overset{P-1}{\underset{j=0}{\sum }}Z(x_{j})e^{-\frac{2\text{π}i}{P}jk}$$

where *L* is the length of the topographic profile, *P* is the number of pixels, Δ*x= L*/*P* is the spatial resolution, *i* is the complex number and *j* and *k* are the discrete indices. The spectral density function *S*(ξ*_k_*) is computed by the Equation (2):

(2) $$S({\text{ξ}}_{k})=\frac{1}{L}{\left|Z({\text{ξ}}_{k})\right|}^{2}$$

The irregularity factor is calculated from the area moments of *S*(ξ*_k_*) when ξ*_k_* > 0. The area moments are obtained by the Equation (3), where Δξ *= L*^−1^ is the spatial frequency resolution. Finally, the Irregularity Factor (*I*) is determined by the Equation (4):

(3) $$M_{n}=\Delta \text{ξ}\overset{P}{\underset{k=0}{\sum }}{\text{ξ}}_{k}^{n}S({\text{ξ}}_{k})$$

(4) $$I=\frac{M_{2}}{\sqrt{M_{0}M_{4}}}$$

A surface with an *I*-Factor close to one has a shape similar to the profile depicted in Figure 1b. The perturbations of the surface are within a narrow frequency band and oscillate respect to the mean plane of the surface. A surface with an *I*-Factor close to zero has a shape similar to the profile shown in Figure 1c. The frequency band is broad, and the amplitude of the perturbations of low spatial frequency is much larger than the perturbations of high spatial frequency.

## 2. Methodology

### 2.1. Materials and Equipment

**Figure 2:**
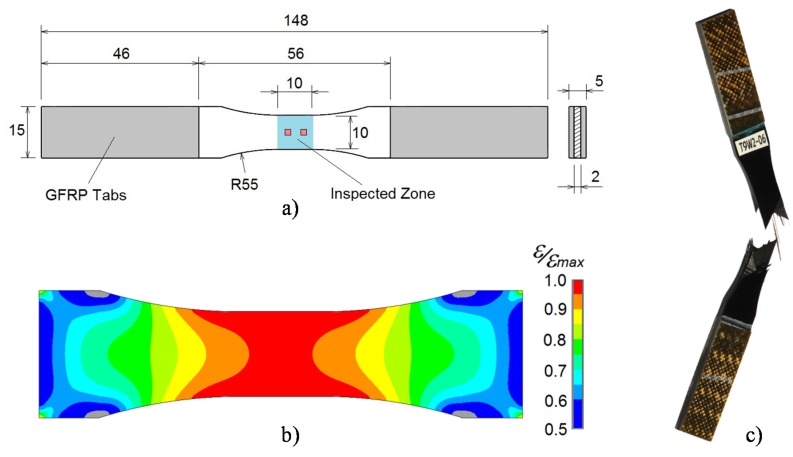
(**a**) Coupon geometry and principal dimensions (mm); (**b**) Normalized strain distribution in the direction of the tensile load by finite element analysis, showing less than 5% of strain gradient in the optical measurement zone; (**c**) Typical failure of a coupon under tensile fatigue loads.

The used composite material is a CFRP type MTM-45-1/IM7 from Cytec Industries Inc. (Heanor, UK), which is a relatively new composite material used for aeronautic structures with the ability to be processed in and out of autoclave. Panels of 2 mm thickness in a quasi-isotropic stacking sequence of ((45,90,−45,0)_s_)_2_, have been autoclave cured at 6 bars and 130 °C for 2.5 h. The ultimate tensile strength (*S_ut_*) of the material is 938 MPa and was determined statistically in a previous study [14]. Coupons with “dog bone” design, shown in Figure 2, were cut from the panels in order to guarantee the highest level of stress at the inspected zone. In order to obtain a better load introduction and to protect the coupons against the gripping forces of the test machine clamps, glass fibre reinforced polymer (GFRP) tabs were bonded at both extremes with film adhesive MTA240 from Cytec Industries Inc.

The fatigue tests were performed with a MTS 810 hydraulic test machine (MTS Systems Co., Eden Prairie, MN, USA) from MTS operated at room temperature under load controlled condition. The stiffness of the specimen was extracted from the test machine during the fatigue cycles. The surface topography was measured by a confocal microscope (PLμ 2300 Confocal Imaging Profiler, from Sensofar (Barcelona, Spain), with an objective zoom of 50×, and a resolution of 5 nm.

### 2.2. Fatigue Tests and Stiffness Measurements

In this work, to study the proposed methodology under different load conditions, three types of fatigue loads were applied. Cycling loads with constant amplitude (CAL), with an *R =* 0.1 which means that the maximum stress (*S_max_*) is 10 times the minimum stress (*S_min_*). Variable amplitude loads (VAL), representative of fighter aircrafts using the standard Falstaff, and VAL representative of transport aircraft using the standard MiniTwist were applied. The quantity of coupons for each type of load and their identification number are summarized in Table 1.

**Table 1:** Quantity of specimens and type of load used for the fatigue test.

Type of Sequence	Number of Coupons	Coupons Id	Tensile Load Ratio *R*
Constant Amplitude Load	13	C01 to C13	0.1
Falstaff	9	F01 to F09	Spectrum *
MiniTwist	5	T01 to T05	Spectrum *

* With a relation of *R =* 0.1 between the maximum peak and minimum valley load.

**Figure 3:**
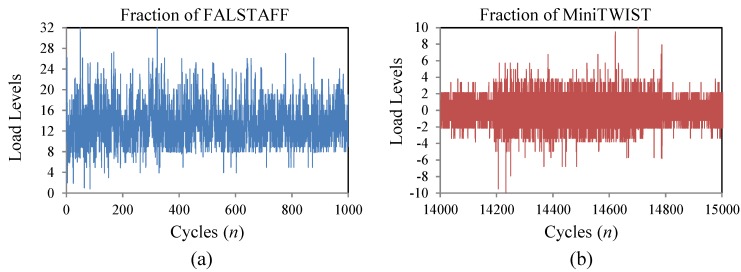
Fraction of the load sequences; (**a**) Fighter Aircraft—Falstaff; (**b**) Transport Aircraft—MiniTwist.

The Falstaff sequence is a standardized representation of the loads supported in the wing roots of fighter aircrafts, under the combinations of different types of missions and maneuvers [23,24]. The complete Falstaff consists of approximately 10^6^ cycles of 32 different load levels which represents 200 flights (one year of typical usage). Figure 3a shows a fraction of the load sequence.

The MiniTwist is a shortened version of the standard Twist (Transport Wing Standard), where minor gust loads were omitted to shorten the tests whilst maintaining the reliability of the results [23,24]. The MiniTwist represents the entire life time fatigue loads in the wing root of transport aircrafts of about 4000 flights and is composed of 580 × 10^3^ cycles discretized in 20 load levels. Figure 3b shows a fraction of the load sequence.

**Figure 4:**
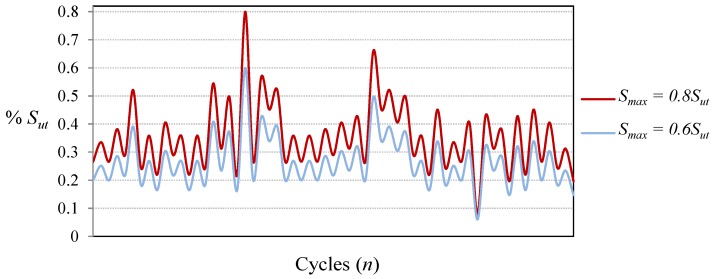
Fraction of Falstaff showing load sequences of different severity, with maximum peak loads of 0.8*S_ut_* and 0.6*S_ut_*.

In order to evaluate the proposed methodology with fatigue cycles of different severity, CALs and VALs with different levels of maximum load have been applied. In the case of Falstaff and MiniTwist, the maximum load is the highest peak in the whole sequence and the minimum load is the lowest valley (in the present study the ratio between the minimum valley and the maximum peak is 0.1). With the aim of obtaining VALs of different severities, the sequences have been scaled to several levels of maximum peak load, as shown in Figure 4.

If a coupon did not fail in whiting one pass of the standard sequences, the load history was repeated until failure with a limit of 10^7^ cycles. In all the cases, the fatigue tests have been interrupted periodically before failure in order to perform the measurements of the surface topography.

The stiffness degradation has been measured during the fatigue cycles using the cross head extension and load data from the testing machine. The stiffness measurements have been normalized to enable them to be quantified in terms of percentage of the initial stiffness.

### 2.3. Measurement of the Surface Irregularity Factor

Measurements of the surface topography have been performed during tests on both coupon sides at an arbitrary location within the center zone of the specimen. Each measurement area is 1.55 × 1.49 mm^2^ with a resolution of the confocal microscope of about 21 megapixels. One value per coupon and per life stage has been obtained by averaging the results of the measured points.

Initial reference topographies were taken previous to the fatigue cycling for each area of measurement, just after the specimen manufacturing. Afterwards, the surface degradation is measured after each fatigue cycling block. Cycling blocks are of arbitrary length and depend on the expected fatigue life of the specimen.

**Figure 5:**
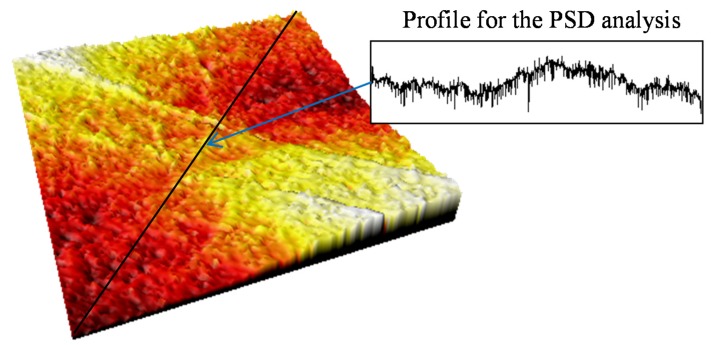
Surface topography in one of the measurement areas and the section profile used to calculate the irregularity factor.

The PSD of each measurement is calculated from a profile extracted from the confocal topography, by sectioning the surface in a plane perpendicular to the reinforcing carbon fibers of the composite material, as shown in Figure 5. The PSD and the surface irregularity factor for each point of measurement are calculated by the Equations (1)–(4).

The evolution of the irregularity factor is evaluated and analyzed in relation to the number of cycles applied and is finally compared with a classical metric of fatigue damage for composite materials, which is the stiffness degradation [5].

## 3. Results and Discussion

### 3.1. Stiffness Degradation

The stiffness degradation is a measure of the damage state of the composite material due to fatigue loads. In this work, it has been studied in relation to the changes of the *I*-factor.

**Figure 6:**
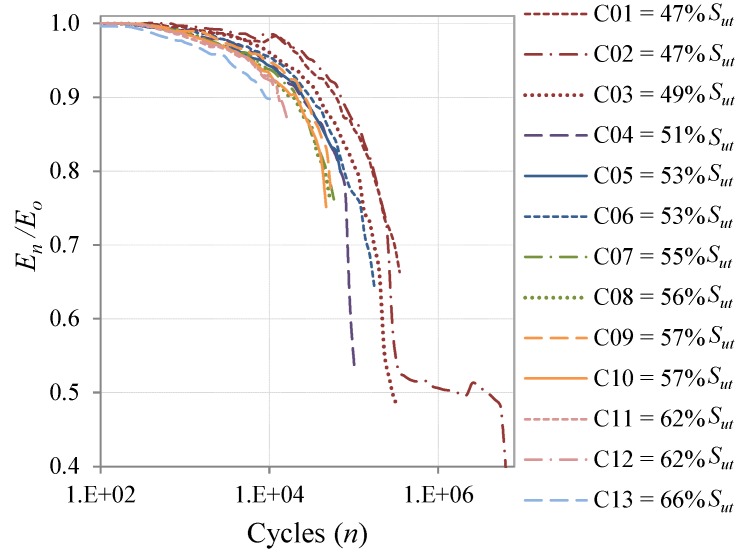
Stiffness degradation of coupons under constant amplitude fatigue loads (CAL) with different maximum load.

**Figure 7:**
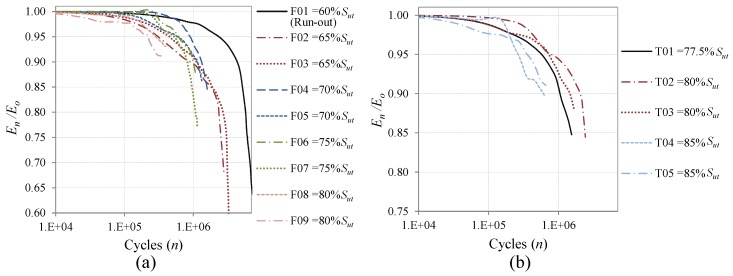
Stiffness degradation of coupons under variable amplitude loads (VAL) with different maximum load; (**a**) Falstaff; (**b**) MiniTwist.

The stiffness degradation as a result of the accumulated damage of the coupons for the CAL tests is shown in Figure 6, and the results for the Falstaff and MiniTwist VAL tests are shown in Figure 7a,b, respectively. All the data of stiffness evolution are presented in semi-log scale. The three graphics show that the coupons cycled with higher severity loads present faster stiffness degradation, because higher loads produce earlier damage in the composite material. This behavior was expected and is in accordance with earlier studies [15,16], and confirms that the stiffness degradation can be employed to determine the damage state due to fatigue loads.

As expected, CAL coupons have failed much earlier at the same maximum load than the VAL coupons, because the maximum loads are only punctually applied in the VAL tests and the majority of the loads are of lower magnitude, whilst in the CAL tests the maximum load is applied cycle by cycle.

Figure 7 also confirms that the Falstaff sequence is more severe than the MiniTwist, because the coupons cycled at the same maximum load failed earlier for Falstaff.

### 3.2. Evolution of the Surface Irregularity Factor (I) through Cycles

**Figure 8:**
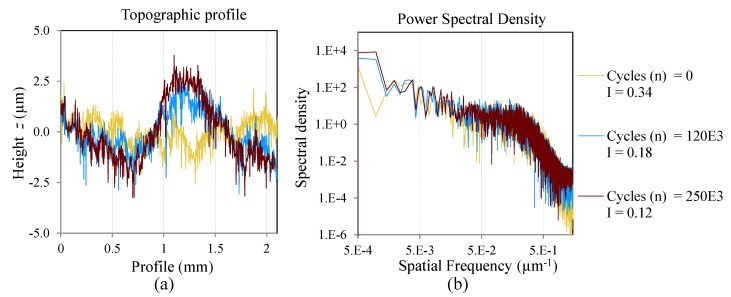
One area of inspection of the coupon C01 cycled at *S_max_* = 0.47*S_ut_*; (**a**) Topographic profile; (**b**) Power spectral density of the profile in log-log scale.

**Figure 9:**
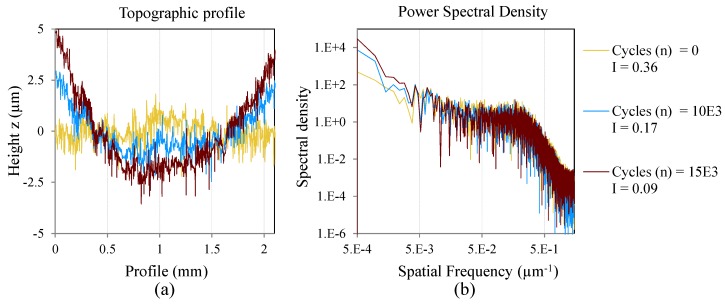
One area of inspection of the coupon C12 cycled at *S_max_* = 0.62*S_ut_* (**a**) Topographic profile; (**b**) Power spectral density of the profile.

The topographic profile and its correspondent spatial spectral density of two measurements and two specimens, are presented to show the phenomena investigated in the present work. The mentioned specimens are cycled with CAL at different maximum loads. Figure 8 and Figure 9 show the topographic profile and the PSD for the coupon C01 cycled at *S_max_* = 47% *S_ut_* and C12 cycled at *S_max_* = 62% *S_ut_*, respectively.

Figure 8a and Figure 9a show the evolution of the topographic profile during the cycles. In both cases a component of low frequency is generated as a consequence of the cracks and delaminations. This low frequency component makes the surface more irregular. With increasing cycles, the amplitude of the low frequency component increases due to an increment in the delamination, and the surface irregularity factor is closer to zero.

Figure 8b and Figure 9b show the evolution of the surface spectral density function. The PSD results are presented in log-log scale. The low frequencies increase due to the aforementioned increment of the delamination and cracks. Also, a variation is shown in the high frequency range where the spectral density increases through the cycles. This variation could be explained by the creation of micro-cracks in the matrix during the fatigue process. These micro-cracks are unrevealed if the topography is analyzed only in the spatial domain.

**Figure 10:**
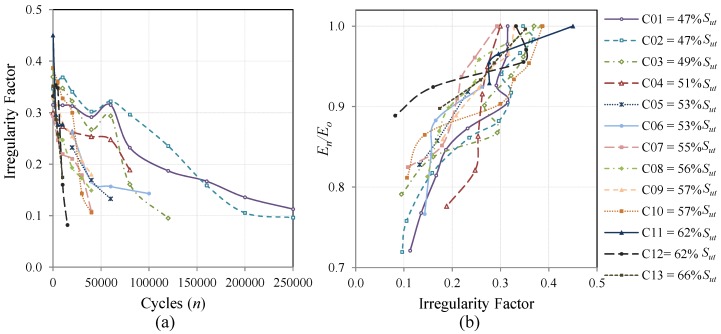
Evolution of the *I*-Factor of all the coupons cycled with CAL. The shown *I*-Factor for each coupon is the mean value of the areas of inspection; (**a**) Evolution through the fatigue cycles; (**b**) Relation between the evolution of the stiffness degradation and the evolution of *I*-Factor.

**Figure 11:**
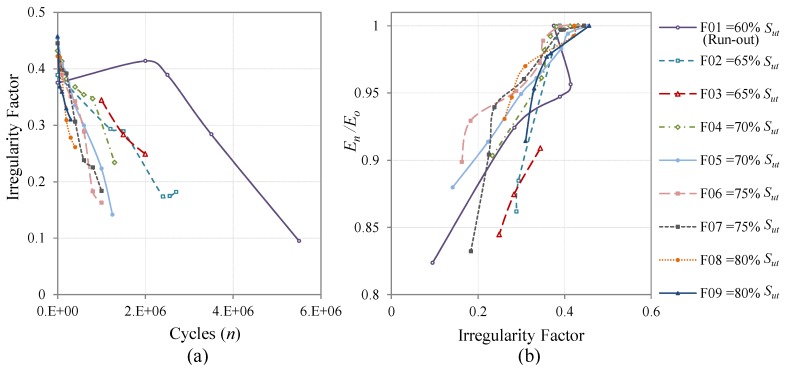
Evolution of the *I*-Factor of all the coupons cycled with Falstaff. The shown *I*-Factor for each coupon is the mean value of all the areas of inspection; (**a**) Evolution through the fatigue cycles; (**b**) Relation between the evolution of the stiffness degradation and the evolution of the *I*-Factor.

**Figure 12:**
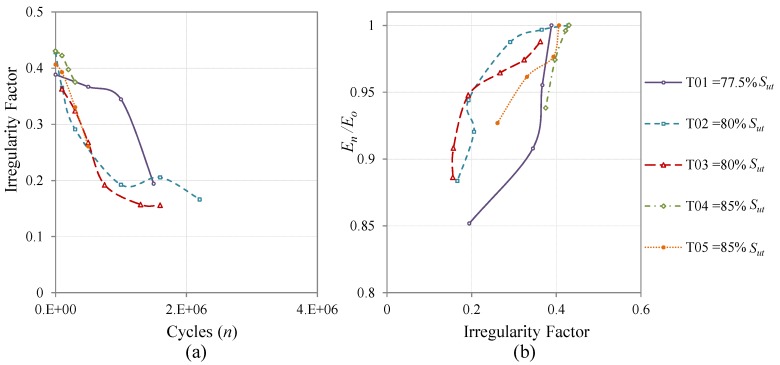
Evolution of the *I*-Factor of all the coupons cycled with MiniTwist. The shown *I*-Factor for each coupon is the mean value of all the areas of inspection; (**a**) Evolution through the fatigue cycles; (**b**) Relation between the evolution of the stiffness degradation and the evolution of the *I*-Factor.

The evolution of the *I*-Factor *versus* the fatigue cycles of all the coupons cycled at CAL is presented in Figure 10a. The specimens’ surfaces become more irregular with increasing fatigue cycles and the slope of the graphs is more pronounced when the fatigue load is higher. The same tendencies can be observed when the coupons are cycled with realistic load sequences. The evolution for Falstaff is presented in Figure 11a and for MiniTwist in Figure 12a. In general, when the load history is of higher severity, a faster change in the irregularity factor is produced. That conclusion is directly related to the evolution of the fatigue damage that higher loads produce more and faster damage than lower loads.

### 3.3. Relation between the Surface Irregularity Factor (I) and the Stiffness Degradation

The stiffness degradation is used in order to establish a direct relation between the surface irregularity factor and the damage of the material due to fatigue.

The relation of the stiffness degradation and the *I*-Factor for CAL tests is depicted in Figure 10b, where the irregularity factor is lower when the stiffness of the material is more degraded. The corresponding Pearson correlation coefficient that indicates the strength of a linear relationship between two variables is 0.84, which confirms the direct relation between the *I*-Factor and the stiffness degradation. The significant dispersion in the results is expected due to the stochastic nature of the fatigue process. However, a clear tendency can be appreciated in the graphic.

Regarding the coupons cycled at realistic load sequences, the same tendency is observed. The linear relation between the *I*-Factor and the stiffness degradation is similar to the CAL tests. The results for Falstaff are shown in Figure 11b with a Pearson correlation of 0.84. The results for MiniTwist are shown in Figure 12b with a Pearson correlation of 0.79. The lower Pearson correlation of the MiniTwist results can be explained by the lower quantity of tested specimens.

**Figure 13:**
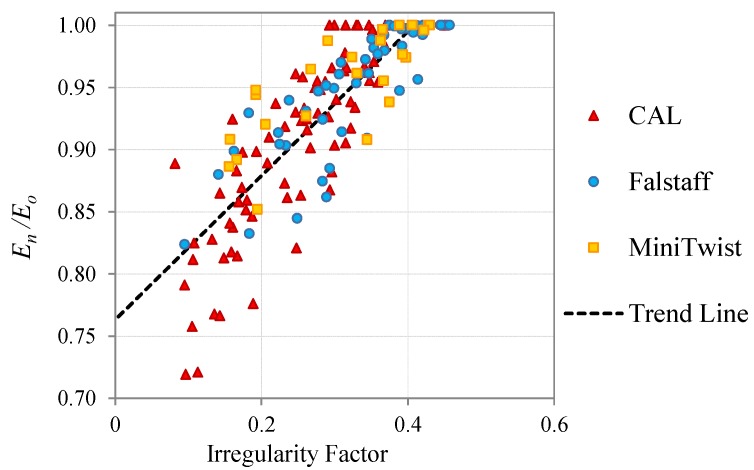
Relation between the stiffness degradation and the *I*-Factor, for the different type of fatigue loads applied.

The relation between the stiffness degradation and the surface irregularity factor for all the load cases studied in the present work (CAL and realistic VAL loads) are presented superimposed in Figure 13. The graph shows that the results present similar tendencies and scattering, which suggest that the present methodology of damage evaluation is independent of the load history that causes the fatigue.

Regarding the initial value it can be seen in the graph that the CAL and the VAL coupons have different *I*-Factor previous to the fatigue process. That is because both groups of coupons are extracted from different panels, and the initial value depends on the manufacturing process of these panels.

From the results presented in Figure 13, we can conclude that the studied methodology can be applied to evaluate the damage state of a structure without knowing the load cases that have caused the surface changes. This makes this technique applicable for a wide range of inspections of composite materials structures from pressurized tanks with CAL fatigue cycles, to variable amplitude loaded aeronautical structures such as wings and empennages, up to automotive and other industrial applications.

## 4. Conclusions

A new inspection technique for the evaluation of the damage state of composite material structures has been presented. It has been demonstrated that the method is applicable for a wide range of fatigue loads, from constant amplitude load cases to complex spectrum loads of fighter and transport aircraft, normalized in Falstaff and MiniTwist standards, respectively. The stiffness degradation and with it, the internal structural damage provoked by fatigue cycles, can be estimated by analyzing and measuring the surface topography using confocal microscopy. Hand held equipment for structural inspections of this type is already on the market. Examples of commercial portable perfilometers from the same manufacturer, and with the same technology and precision as the confocal microscope employed to obtain the results of the present work, are the models PLμ 1300 and Smart, both from Sensofar.

The surface irregularity factor, as a parameter to measure the damage state of a carbon fiber component, shows a good agreement with a classical damage metric such as the stiffness degradation. To consider the Pearson correlation of 0.84 as good, we should take into account that fatigue processes are a stochastic phenomenon with high variability.

Analysis in the spatial frequency domain could reveal surface changes due to fatigue damage that are hidden in an analysis of the surface roughness magnitude [15,16]. Internal delaminations are revealed by low spatial frequency components and cracks and micro-cracks increase the high spatial frequency content.

The results obtained with the present methodology are independent of the load type applied and can be used for a wide range of applications where the in-service loads are known and controlled (such as pressure vessels) and where the loads are of random or variable nature (wings, automotive components, *etc.*).

## References

[1] Boller C., Staszewski W., Staszewski W., Boller C., Tomlinson G. (2004). Aircraft Structural Health and Usage Monitoring. Health Monitoring of Aerospace Structures: Smart Sensors Technologies and Signal Processing.

[2] Post N.L., Case S.W., Lesko J.J. (2008). Modeling the variable amplitude fatigue of composite materials: A review and evaluation of the state of the art for spectrum loading. Int. J. Fatigue.

[3] Epaarachchi J.A. (2006). A study on estimation of damage accumulation of glass fibre reinforce plastic (GFRP) composites under a block loading situation. Compos. Struct..

[4] Zuluaga-Ramírez P., Frövel M., Arconada Á., Belenguer T., Salazar F. (2014). Evaluation of the fatigue linear damage accumulation rule for aeronautical CFRP using artificial neural networks. Adv. Mater. Res..

[5] Highsmith A.L., Reifsnider K.L., Reifsnider K.L. (1982). Stiffness Reduction Mechanisms in Composite Laminates. Damage of Composite Materials.

[6] Whitworth H.A. (1997). A stiffness degradation model for composite laminates under fatigue loading. Compos. Struct..

[7] Van Paepegem W., Degrieck J. (2002). Coupled residual stiffness and strength model for fatigue of fibre-reinforced composite materials. Compos. Sci. Technol..

[8] Dzenis Y.A. (2003). Cycle-based analysis of damage and failure in advanced composites under fatigue: 1. Experimental observation of damage development within loading cycles. Int. J. Fatigue.

[9] Giancane S., Panella F.W., Nobile R., Dattoma V. (2010). Fatigue damage evolution of fiber reinforced composites with digital image correlation analysis. Proced. Eng..

[10] Dattoma V., Giancane S. (2013). Evaluation of energy of fatigue damage into GFRC through digital image correlation and thermography. Compos. B Eng..

[11] Ahsan M., Han X., Islam S., Newaz G. (2004). Fatigue damage detection in graphite/epoxy composites using sonic infrared imaging technique. Compos. Sci. Technol..

[12] Withers P.J., Preuss M. (2012). Fatigue and damage in structural materials studied by X-ray tomography. Annu. Rev. Mater. Res..

[13] Wang X., Chung D.D.L. (1998). Self-monitoring of fatigue damage and dynamic strain in carbon fiber polymer-matrix composite. Compos. B Eng..

[14] Zuluaga P., Frövel M., Restrepo R., Trallero R., Atienza R., Pintado J.M., Belenguer T., Salazar F. (2013). Consumed fatigue life assessment of composite material structures by optical surface roughness inspection. Key Eng. Mater..

[15] Zuluaga-Ramírez P., Frövel M., Belenguer T., Salazar F. (2015). Non-contact inspection of the fatigue damage state of carbon fiber reinforced polymer by optical surface roughness measurements. NDT E Int..

[16] Zuluaga-Ramírez P., Arconada Á., Frövel M., Belenguer T., Salazar F. (2015). Optical sensing of the fatigue damage state of CFRP under realistic aeronautical load sequences. Sensors.

[17] Gordienko Y.G., Zasimchuk E.E., Gontareva R.G. (2002). Unconventional deformation modes and surface roughness evolution in Al single crystals under restricted cyclic tension conditions. J. Mater. Sci. Lett..

[18] Wang L., Wang Z., Xie W., Song X. (2012). Fractal study on collective evolution of short fatigue cracks under complex stress conditions. Int. J. Fatigue.

[19] Sonsino C.M. (2007). Fatigue testing under variable amplitude loading. Int. J. Fatigue.

[20] Rice S.O. (1944). Mathematical Analysis of Random Noise. Bell Syst. Tech. J..

[21] Rice S.O. (1945). Mathematical Analysis of Random Noise. Bell Syst. Tech. J..

[22] Petrucci G., Zuccarello B. (2004). Fatigue life prediction under wide band random loading. Fatigue Fract. Eng. Mater. Struct..

[23] Engineering Sciences Data Unit (ESDU) (1999). IHS ESDU 97018, Standard Fatigue Loading Sequences.

[24] Heuler P., Klätschke H. (2005). Generation and use of standardised load spectra and load-time histories. Int. J. Fatigue.

